# Expression and localization of GPR109A (PUMA-G/HM74A) mRNA and protein in mammalian retinal pigment epithelium

**Published:** 2009-02-16

**Authors:** Pamela M. Martin, Sudha Ananth, Gail Cresci, Penny Roon, Sylvia Smith, Vadivel Ganapathy

**Affiliations:** 1 Department of Biochemistry and Molecular Biology, Medical College of Georgia, Augusta, GA; 2 Department of Cellular Biology and Anatomy, Medical College of Georgia, Augusta, GA; 3 Department of Ophthalmology, Medical College of Georgia, Augusta, GA; 4 Department of Surgery, Medical College of Georgia, Augusta, GA

## Abstract

**Purpose:**

GPR109A has been identified as a G-protein-coupled receptor for niacin. β-hydroxybutyrate (β-HB) is a physiologic ligand for the receptor. β-HB, the predominate ketone body in circulation, is an important energy source for neurons, including retinal neurons, under various physiologic and pathologic conditions. The identification of GPR109A as the receptor for β-HB suggests additional, hitherto unknown, functions for this metabolite. The circulating levels of β-HB increase in diabetes. Since retinopathy is a serious complication associated with diabetes, we investigated GPR109A expression in retina and in different retinal cell types to determine if the receptor may have a role in the pathophysiology of diabetic retinopathy.

**Methods:**

RT–PCR, fluorescent in situ hybridization, and immunofluorescent techniques were used to analyze GPR109A expression in mouse retina and in three transformed retinal cell lines: ARPE-19 (RPE), RGC-5 (ganglion), and rMC-1 (Müller). Activation of GPR109A by niacin and β-HB was demonstrated in ARPE-19 cells by cAMP assay.

**Results:**

Studies conducted using mouse retinal tissues demonstrated that GPR109A is expressed in retina with its expression restricted to RPE, where it differentially polarizes to the basolateral membrane. These results were confirmed with cell lines, which demonstrated GPR109A expression in ARPE-19, but not in rMC-1 and RGC-5 cells. Primary cultures of mouse RPE also showed robust expression of GPR109A. cAMP assay demonstrated that GPR109A expressed in RPE is functional.

**Conclusions:**

These data represent the first report on GPR109A expression in retina. The exclusive expression of GPR109A in RPE basolateral membrane, which has access to β-HB in blood, may have biologic importance in diabetic retinopathy.

## Introduction

G protein-coupled receptors (GPCRs) comprise a large protein family of transmembrane receptors that play pivotal roles in several biologic processes. As such, GPCRs are currently the target of several pharmaceuticals aimed at treating pain, inflammation, and a broad range of diseases including cancer, cardiac disease, and diabetes [1,2]. Of the more than 700 GPCRs identified by the Human Genome Project, 150 or more of these receptors are annotated as “orphan” or functionally uncharacterized [3]. Prior to its identification as the nicotinic acid (niacin) receptor [4-6], GPR109A was classified also as an orphan GPCR. GPR109A is expressed primarily in adipocytes and immune cells. Indeed, it is the abundant expression of the receptor in adipocytes that led to its identification as the receptor for niacin, a drug widely used for decades in the treatment of hyperlipidemia. GPR109A is coupled to the inhibitory G protein G_i_. Activation of the receptor with its ligands results in a pertussis toxin-sensitive decrease in cAMP levels. One of the actions of cAMP in adipocytes is to stimulate the hormone-sensitive lipase by protein phosphorylation, which leads to triglyceride hydrolysis and release of free fatty acids into the circulation. Niacin, by its ability to reduce cAMP levels, decreases the activity of lipase and prevents the release of free fatty acids from fat stores.

While the discovery of GPR109A as a receptor for niacin helps to explain the mechanism of action of the drug, it sheds little light on any physiologic function of the receptor, as circulating levels of niacin in normal individuals not taking the drug for therapeutic purposes are too low to activate the receptor [5,7]. This led to the identification of β-hydroxybutyrate (β-HB) as the physiologic ligand for the receptor [8]. As with niacin, interaction of β-HB with the receptor decreases intracellular cAMP levels. Since β-HB is generated in the liver from fatty acid oxidation following fat mobilization from adipocytes, it is believed that β-HB, through its interaction with GPR109A present on adipocytes, may inhibit triglyceride hydrolysis and release of free fatty acids as a negative feedback regulatory mechanism, promoting the maintenance of metabolic homeostasis during conditions such as starvation and diabetes [8].

Though GPR109A is most noted for its anti-lipolytic effects in adipocytes, recent studies demonstrate that the activation of the receptor produces differential responses depending on the location/cell-type in which the receptor is expressed. For example, activation of GPR109A on dermal dendritic cells or dermal macrophages mediates the characteristic cutaneous vasodilation or “flushing” response associated with chronic therapeutic use of niacin, an effect very different from the antilipolytic actions seen in adipocytes [9]. In murine macrophages, GPR109A expression is upregulated by cytokines such as interferon-gamma (IFN-γ) [10], suggesting a role for GPR109A in immunity and inflammation. Thus, GPR109A expression and function likely extends beyond adipocytes and immune cells.

In the present study, we asked whether GPR109A is expressed also in retina. We were interested in expression of the receptor in this tissue for several reasons. First, chronic use of niacin at high doses necessary for therapeutic efficacy as a lipid-lowering agent is associated with several side effects, including an ocular complication known as niacin maculopathy [11-13]. This suggests that the niacin receptor GPR109A may be present also in retina and contribute to the pathogenesis of maculopathy. Second, activation of GPR109A is associated with decreased cAMP levels. The essential role of cAMP in maintenance of neuronal health in retina and overall visual function is widely accepted, as alterations in cAMP levels severely compromise retinal function [14-17]. Thus, if GPR109A is expressed in retina, it is possible that alterations in cAMP levels as a consequence of GPR109A activation might play a role in these vital retinal processes. Under normal physiologic conditions, in the absence of niacin drug therapy, this would likely be of little relevance; β-HB, the predominant ketone body in circulation and endogenous ligand for GPR109A, is normally found at low levels in the circulation (<50 μM), concentrations insufficient to activate the receptor (concentrations necessary to elicit half-maximal effect on the receptor, 300–800 μM). β-HB levels increase several-fold (approximately 3 mM), however, during periods of prolonged fasting, starvation, and strenuous exercise to maintain the energy status in vital organs, including brain and retina. The same is true in poorly controlled diabetes (especially type 1 diabetes), when circulating levels of β-HB may increase to as much as roughly 20 mM [18]. Equally interesting is the postulated role for GPR109A in the inflammatory response in certain cell types [10,19,20]. Increasing evidence points to the involvement of inflammatory processes in the pathogenesis of several retinal diseases, including diabetic retinopathy and macular degeneration [19,20]. Thus, determining whether GPR109A is expressed in retinal cells and understanding how the receptor is regulated in retina would be of immense clinical relevance, particularly in diabetic conditions when levels of β-HB are substantially elevated.

## Methods

### Reagents

Reagents used in cell culture, retinal pigment epithelium (RPE) cell isolation, and RNA extraction, Hoechst 33342, Image-iT FX signal enhancer, and secondary antibodies were from Invitrogen (Carlsbad, CA). GeneAmp RT–PCR kit was from Applied Biosystems, Inc. (Foster City, CA). DIG RNA Labeling Kit was from Roche (Indianapolis, IN). *Taq* polymerase kit was from either TaKaRa (Tokyo, Japan) or Biogenex (San Ramon, CA). cAMP assay kit was from Assay Designs, Inc. (Ann Arbor, MI). Primary antibodies were obtained from the following sources: peroxidase-conjugated monoclonal anti-digoxigenin antibody (Jackson ImmunoResearch, West Grove, PA); anti-mouse tyramide signal amplification (TSA)-Cy3 conjugate (Perkin Elmer, Wellesley, MA); mouse monoclonal anti-cellular retinaldehyde binding protein (CRALBP), and mouse monoclonal anti-RPE65 antibody (AbCam, Cambridge, MA); chicken polyclonal anti-monocarboxylate transporter 1 (MCT1; Chemicon International, Temecula, CA); Vectashield Hardset Mounting Media (Vector Laboratories, Burlingame, CA); [^3^H]nicotinate (specific radioactivity, 30 Ci/mmol; American Radiolabeled Company, Inc., St. Louis, MO).

### Generation of anti-GPR109A antibody

A rabbit polyclonal antibody against GPR109A was generated commercially (Genscript Corp., Piscataway, NJ). The antibody was raised against the peptide sequence RKKTLGEPDNNRSTSV, which corresponds to residues 311–326 of mouse GPR109A. The corresponding sequence in rat GPR109A is RRKTLGEPDNNRSTSV, which differs from the antigenic peptide in only one amino acid. The corresponding sequence in human GPR109A is QRKMTGEPDNNRSTSV, which differs from the antigenic peptide in four amino acids. We investigated the species specificity of the antibody and found that the antibody recognizes GPR109A in all three species. Rodents have a single gene coding for GPR109A, whereas humans have two closely related genes coding for highly homologous receptors (GPR109A and GPR109B) [2]. Even though the amino acid sequences of GPR109A and GPR109B are similar, GPR109B does not function as a niacin or β-hydroxybutyrate receptor. Since the region containing the antigenic peptide is identical in GPR109A and GPR109B, the antibody is expected to recognize not only GPR109A but also GPR109B in human tissues or cell lines.

Prior to use in immunohistochemical studies, the specificity of the antibody was confirmed by analysis of antibody staining in a cell line that does not express GPR109A constitutively. Briefly, rat, mouse, or human GPR109A was expressed in cells via lipofectamine-mediated transient transfection (transfection efficiency >90%). Immunofluorescence experiments were then performed in parallel in nontransfected cells and in GPR109A-transfected cells. Nontransfected cells served as a negative control. Normal rabbit pre-immune serum was used as an additional negative control, as was peptide-blocked antigenic peptide. For peptide blocking experiments, the anti-GPR109A antibody was neutralized with 1 μg/ml of the antigenic peptide and used as a negative control for immunofluorescence studies. No immunopositive signal was detected when the antibody was used in nontransfected cells, cells incubated with pre-immune serum, or peptide-blocked antigenic peptide (data not shown). However, the antibody gave strong positive signal in cells transfected with the rat, mouse, or human GPR109A construct.

### Animals

The present study used 3-week-old C57BL/6 mice for the preparation of total RNA from neural retina and RPE/eyecup, and for the establishment of primary RPE cell cultures. Albino (Balb/c) mice, age 6–9 weeks, were used for immunofluorescence and fluorescent in situ hybridization analyses. All animals were purchased from Harlan (Indianapolis, IN). Mice were maintained in clear plastic cages and subjected to standard light cycles (12 h light/12 h dark). Mice were fed Harlan’s Teklad rodent diet #8604 (min. crude protein, 24.0%; min. crude fat, 4.0%; max. crude fiber, 4.5%) and water was available ad libitum. Care and use of the mice adhered to the principles set forth in the Public Health Service Policy on Humane Care and Use of Laboratory Animals.

### Cell lines

A human retinal pigment epithelial cell line (ARPE-19), rat ganglion cell line (RGC-5), and rat Müller cell line (rMC-1) were cultured in Dulbecco’s modified Eagle medium (DMEM)/F12 medium and maintained at 37 °C in a humidified chamber of 5% CO_2_. All culture media were supplemented with 10% fetal bovine serum, 100 U/ml penicillin, and 100 μg/ml streptomycin. The culture medium was replaced with fresh medium every other day. Upon confluency, cultures were passaged by dissociation in 0.05% (w/v) trypsin in phosphate-buffered saline (PBS; 0.01 M phosphate buffer, 0.0027 M KCl, 0.137 M NaCl, pH 7.4). For immunocytochemistry, ARPE-19, RGC-5, or rMC-1 cells were cultured on glass coverslips for 1–2 days; ARPE-19 cells were not polarized.

### Establishment of primary RPE cell cultures from mouse eyes

C57Bl/6 mice were used for preparation of primary RPE cell cultures according to our previously published method [21]. Briefly, enucleated mouse eyes were rinsed in 5% povidone-iodine solution, followed by rinsing with sterile Hank’s Balanced Salt Solution (HBSS). Eyes were then placed in cold RPE cell culture medium (DMEM:F12), supplemented with 25% fetal bovine serum, 0.1 mg/ml gentamicin, 100 U/ml penicillin, and 100 µg/ml streptomycin. To aid in the degradation of extracellular matrix components and enhance the dissociation of RPE from neural retina and choroid, eyes were then incubated in HBSS, containing 19.5 U/ml collagenase and 38 U/ml testicular hyaluronidase for 40 min at 37 °C, followed by incubation in HBSS containing 0.1% trypsin (pH 8) for 50 min at 37 °C. Eyes were then dissected to separate RPE from neural retina. Isolated RPE cells were collected in a 15 ml centrifuge tube and centrifuged at at 1,228 × g for 10 min (Thermo Medilite Centrifuge, ThermoScientific, Waltham, MA), followed by resuspension in RPE cell media. RPE cells were then cultured at 37 °C. The purity of RPE cells was evaluated by the expression of CRALBP (a marker for RPE cells and Müller cells) and RPE-65 (a marker for RPE cells) using specific antibodies.

### RT–PCR

Neural retina and RPE/eyecup were prepared according to our previously published method [22] and used for preparation of total RNA. Briefly, the eye was proptosed and the cornea slit which immediately released the lens (and vitreous). The retina was then dissected away from the remaining RPE-choroid-eyecup complex. Tissue from six eyes were pooled for each analysis and total RNA was prepared. Total RNA was also prepared from three transformed retinal cell lines: rMC-1, RGC-5, and ARPE-19. Primary RPE cells isolated from mouse eyes (mRPE) were also used for RNA isolation. RT–PCR was performed using primer pairs specific for mouse, rat, or human GPR109A (Table 1). 18S rRNA or hypoxanthine guanine phosphoribosyltransferase (HPRT) was used as an internal control for the PCR reaction. The products were subcloned into the pGEM-T vector and sequenced to confirm their molecular identity.

**Table 1:** Sequences of RT–PCR primers.

**Gene name**	**Primer sequence**	**Expected product size (bp)**
Human GPR109A (HM74A)	F: GGACAACTATGTGAGGCGTTGG	650
	R: GGGCTGGAGAAGTAGTACACC	
Human GPR109B (HM74)	F: CGTGATGGACTACTATGTGCG	280
	R: ATTTGCAGGGCCATTCTGGAT	
Mouse GPR109A (mPUMA-G)	F: GTTACAACTTCAGGTGGCACGAT	443
	R: CTCCACACTAGTGCTTCGGTTATT	
Rat GPR109A (rPUMA-G)	F: ACTTTCTGGTGATAAACGGCAAGA	455
	R: GACTGTCAGGCCGATGGTG	
HPRT1	F: GCGTCGTGATTAGCGATGATGAAC	150
	R: CCTCCCATCTCCTTCATGACATCT	
18S	Universal Standard purchased from Applied Biosystems/Ambion (Austin, TX) Cat #: AM1716	315

Total RNA was prepared from intact retinal tissues or transformed cell lines and used for analysis of GPR109A expression by RT–PCR. The specific primer sequences as well as the expected product size for all of the primers used in this study are provided in this table. All PCR reactions were performed for 35 cycles at an annealing temperature of 58 °C.

### Fluorescent in situ hybridization

To localize the mRNA transcript encoding GPR109A in intact mouse retina, we prepared retinal cryosections by following our previously published method and used these sections for fluorescent in situ hybridization (FISH) analysis [21,22]. The methods of Mullis and Faloona [23] and Stoflet et al. [24] were employed to generate digoxigenin (DIG)-labeled riboprobes, using the aforedescribed PCR primers for RT–PCR analysis. Briefly, the 23 bp T7 promoter sequence (5′-TAA TAC GAC TCA CTA TAG GGA G-3′) was appended to the 5′ end of the antisense primer such that the promoter was incorporated into the PCR product. Subsequent amplification of the target DNA (cDNA prepared using RNA from mouse RPE/eyecup) yielded a PCR product that contained the T7 promoter sequence upstream of the mouse GPR109A sequence. To produce a template for transcription of the sense (control) strand of the amplified region, we designed an additional primer set with the T7 promoter sequence added to the sense-strand GPR109A primer. These templates were then used for the synthesis of riboprobes (antisense and sense) with T7 RNA polymerase. A DIG tag for antibody labeling was added to sense and antisense probes using a DIG RNA labeling kit (Roche). Mouse eyes were frozen immediately in Tissue-Tek Optimal Cutting Temperature compound (OCT; Sakura Finetek, Torrance, CA), and sections were made at 10-µm thickness and fixed in 4% paraformaldehyde for 30 min at room temperature. Sections were rinsed in ice-cold PBS and treated with 0.1% diethylpyrocarbonate prepared in PBS to facilitate penetration of the labeled probe. Sections were permeabilized further with 1 mg/ml proteinase K in PBS for 4 min. The proteinase K activity was stopped by rinsing the slides in 2 mg/ml glycine in PBS. Sections were washed in PBS, equilibrated in 5% saline-sodium citrate (SSC), and prehybridized for 2 h at 58 °C in 50% formamide, 5% SSC, and 50 µg/ml salmon sperm DNA (pH 7.4). Sections were hybridized with 1 µg/ml DIG-labeled antisense or sense probes and incubated overnight at 58 °C. The sections were washed under high stringency conditions, first in 2% SSC for 1 h at 65 °C, and then in 0.1% SSC for 1 h at 65 °C, and transferred to PBS. Immunologic detection of the probe was performed by blocking sections with PBS-BB (PBS + 1% BSA, 0.2% powdered skim milk, and 0.3% Triton X-100) for 30 min at room temperature. DIG-labeled probes were detected by incubating the sections with 1:1000 peroxidase-conjugated mouse monoclonal anti-digoxigenin antibody for 1 h at room temperature. Sections were then washed in PBS, followed by incubation with 1:500 anti-mouse TSA-Cy3 conjugate 30 min at room temperature. Next, 1:10,000 Hoechst 33342 was used to counterstain the cell nuclei, and coverslips were mounted using Vectashield Hardset mounting medium. Sections were examined using a Zeiss Axioplan 2 fluorescent microscope equipped with an ApoTome, AxioVision 4.5 software, and an HRM camera (Carl Zeiss, Oberkochen, Germany).

### Fluorescence immunohistochemistry

To localize GPR109A protein in intact retina with respect to known markers of RPE apical and basolateral membranes, we employed immunofluorescent methods. Cryosections of mouse eyes were fixed in 4% paraformaldehyde for 10 min, washed with PBS, and blocked with 1X Power Block for 10 min at room temperature. Sections were then incubated overnight at 4 °C with 1:250 rabbit polyclonal anti-GPR109A antiserum. Double-labeling studies were performed with 1:1,000 chicken polyclonal anti-MCT1 as a positive marker for RPE apical membrane [25,26]. Negative control sections were treated identically except that primary GPR109A antiserum, neutralized by incubation with excess antigenic peptide (single-labeling experiments) or PBS (double-labeling experiments), was substituted in place of primary antibodies for overnight incubation. Sections were rinsed with PBS and incubated for 45 min at room temperature with secondary antibodies. For detection of GPR109A labeling, sections were incubated with 1:1,000 goat anti-rabbit Alexa Fluor 568. Sections double-labeled with GPR109A and MCT1 antibodies were incubated with 1:500 goat anti-rabbit Alexa Fluor 488 and 1:1,000 goat anti-chicken Alexa Fluor 568, respectively.

Immunofluorescent methods were used to localize GPR109A protein in cultured ARPE-19, rMC-1, RGC-5, and primary mouse RPE cells, grown on glass coverslips. Cells were air-dried to facilitate better adhesion during the experiment, and fixed with 4% paraformaldehyde for 5 min at room temperature. Cells were then washed with PBS and blocked with Image-iT signal enhancer for 30 min. Incubation of cells with 1:100 anti-GPR109A-specific primary antibody was performed overnight at 4 °C. Cells were again washed in PBS and incubated with 1:1,000 goat anti-rabbit Alexa Fluor 568 for 45 min at room temperature. Nuclei were counterstained with 1:10,000 Hoechst 33342 and coverslips inverted and mounted on glass slides using Vectashield Hardset mounting medium.

### cAMP measurement

To determine whether GPR109A expressed on RPE cells was functional, we analyzed the effect of the GPR109A ligands nicotinic acid and β-HB on intracellular cAMP levels in ARPE-19 cells. Briefly, ARPE-19 cells were seeded at a density of 0.1×10^6^ cells/well in a 24 well plate. On the third day after seeding, culture medium was removed and replaced with culture medium containing 10 μM forskolin in the presence or absence of 1 mM nicotinic acid or β-HB for 1 h. Measurement of intracellular levels of cAMP was performed using a commercially available kit and following the manufacturer’s instructions. Experiments were repeated three times with independent cell cultures, and cAMP measurements were made in triplicate in each experiment. Data are presented as means±SEM from the three separate experiments.

## Results

### GPR109A mRNA expression in mouse retina

**Figure 1:**
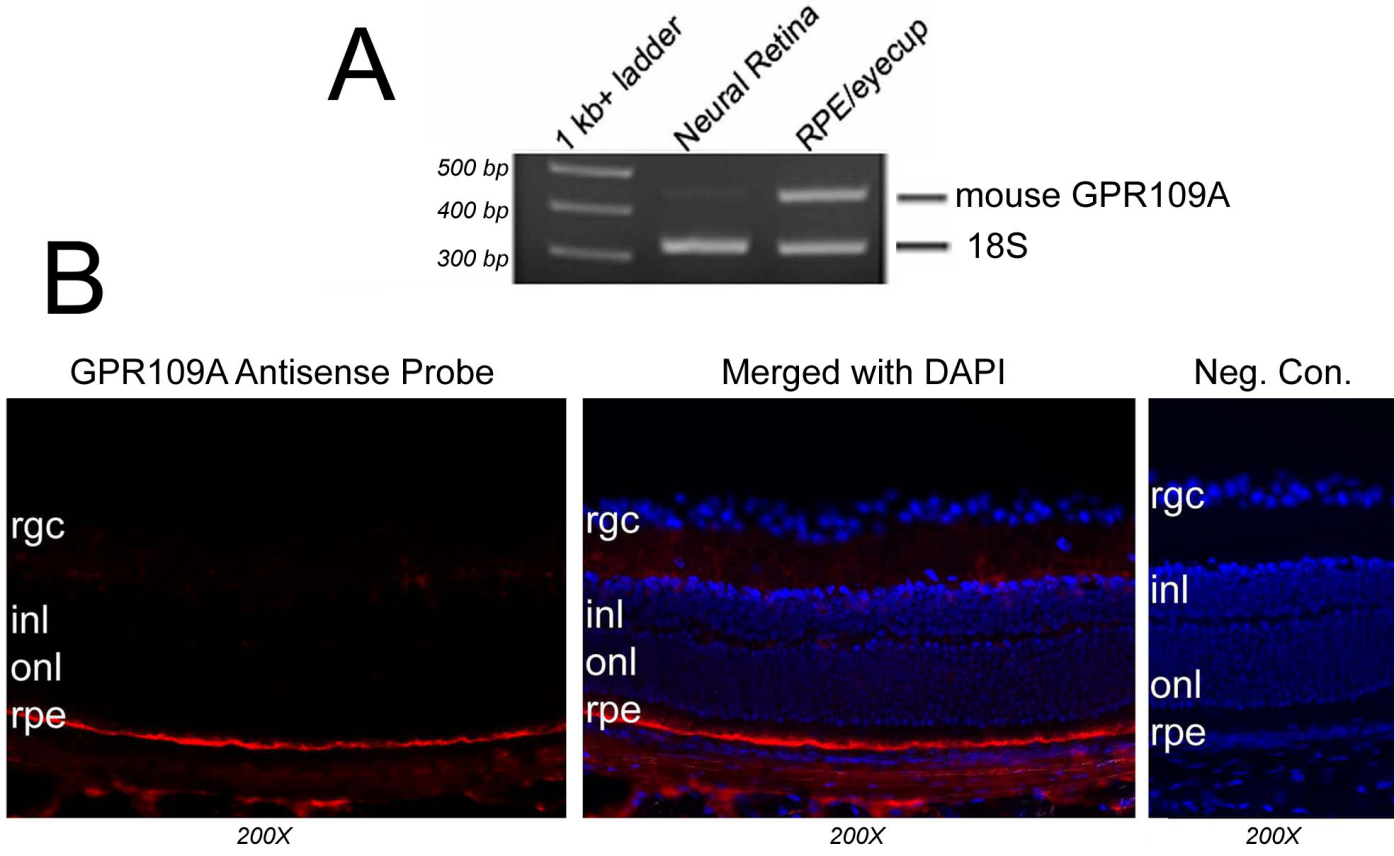
GPR109A mRNA expression in mouse retina. **A:** Total RNA collected from mouse neural retina and retinal pigment epithelium (RPE)/eyecup was used for RT–PCR analysis of GPR109A mRNA expression. GPR109A expression was detected in RPE/eyecup but not in neural retina. 18S rRNA was used as an internal control. **B:** Localization of GPR109A mRNA transcripts was evaluated by fluorescent in situ hybridization (FISH). Hybridization of mouse retinal cryosections with GPR109A-specific antisense probe detected positive (red) labeling indicative of GPR109A expression in the RPE. No positive signal was detected in sections hybridized with sense (negative control) probe. Abbreviations: retinal ganglion cell layer (rgc); inner nuclear layer (inl); outer nuclear layer (onl). Magnification of all images in panel **B** is 200X.

To determine whether mRNA transcripts encoding GPR109A were present in mouse retina, we used RNA isolated from RPE/eyecup and neural retina for RT–PCR (Figure 1A). PCR was performed using a primer pair specific for mouse GPR109A (Table 1). 18S rRNA was used as an internal control. GPR109A mRNA expression was detected in RPE/eyecup, but not in neural retina. As expected, the actual size of the PCR product was 443 bp. The molecular identity of the product from RPE/eyecup was confirmed by sequencing. The expression pattern of GPR109A mRNA in intact retina was then analyzed by FISH using DIG-labeled antisense riboprobe specific for mouse GPR109A (Figure 1B). Hybridization of retinal cryosections with GPR109A antisense probe gave a positive fluorescence (red) signal detectable only in the RPE cell layer (Figure 1B). The fluorescent signal is specific as evident from the lack of positive signal in sections hybridized with sense riboprobe (negative control).

### Localization of GPR109A protein in mouse retina

**Figure 2:**
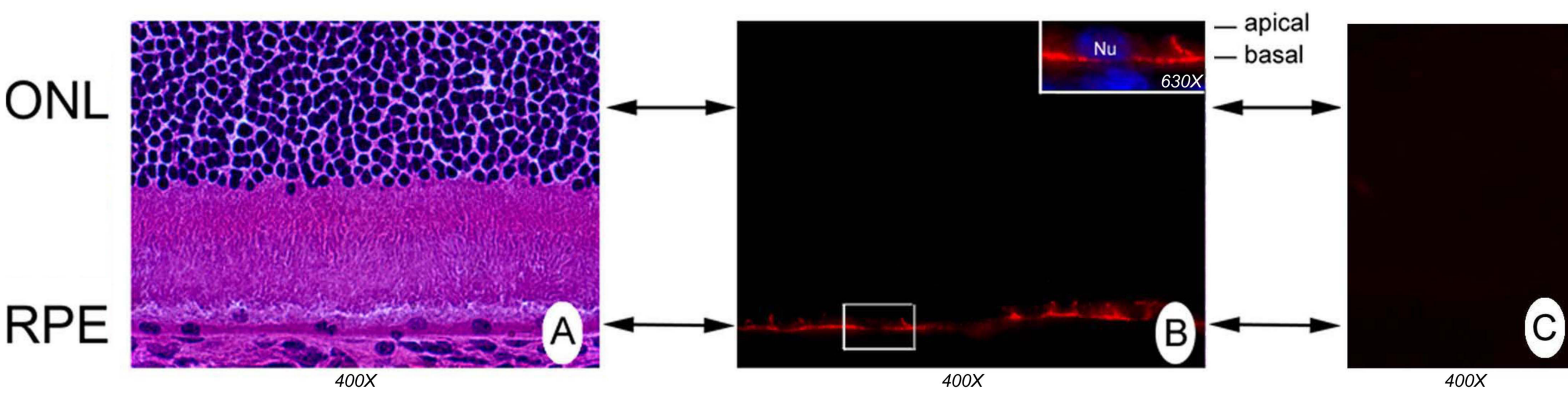
Localization of GPR109A protein in intact retina. **A:** A hematoxylin and eosin-stained retinal cryosections is provided for comparison to panel **B** of the figure. **B:** Incubation of mouse retinal cryosections with anti- GPR109A antibody revealed positive (red) labeling for GPR109A protein exclusively in retinal pigment epithelium (RPE). Higher magnification of cryosections reveals that the GPR109A protein is associated specifically with RPE basolateral membrane (panel **B,** inset). Hoechst 33342 nuclear stain is shown in blue. In the inset, Nu means nucleus. **C:** No labeling was detected in retinal cryosections incubated with primary GPR109A antibody neutralized by incubation with excess antigenic peptide.

**Figure 3:**
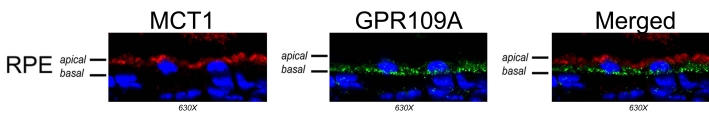
Double-labeling immunofluorescence techniques were employed to determine whether GPR109A protein is expressed apically or basolaterally in RPE. Chicken polyclonal anti-monocarboxylate transporter 1 (MCT1) was used as a positive marker for RPE apical membrane. Sections were then viewed and optically sectioned using a Zeiss Axioplan fluorescent microscope equipped with an apotome. Merging of positive signals for MCT1 (red) and GPR109A protein (green) did not display any significant overlap. Hoechst 33342 nuclear stain is shown in blue.

RT–PCR and FISH analyses demonstrated the presence of GPR109A mRNA in mouse retina, with expression restricted exclusively to the RPE. To determine whether GPR109A protein itself could be detected in mouse retina, we performed immunofluorescence analysis using an antibody specific for GPR109A (Figure 2 and Figure 3). Incubation of retinal cryosections with the antibody gave a positive fluorescence (red) signal only in the RPE cell layer (Figure 2B). A corresponding hematoxylin- and eosin-stained retinal cryosection is shown in Figure 2A for comparison. Retinal cryosections incubated with primary GPR109A antibody neutralized by incubation with excess antigenic peptide showed no positive signal (Figure 2C). Observation of the RPE cell layer at higher magnification (Figure 2B, inset) indicated that positive labeling of GPR109A protein appeared to be associated specifically with the basolateral membrane of this polarized cell. Differential distribution of GPR109A to the basolateral membrane of RPE, a location conducive to interaction of the receptor with ligands present in the choroidal circulation, may have important implications in terms of the functional role of this receptor in retina. Therefore, to confirm the polarized localization of GPR109A to the basolateral membrane of RPE, we performed additional experiments. Retinal cryosections were stained with GPR109A antibody along with antibody against MCT1 [25,26]. Immunofluorescence analysis of these sections using a Zeiss Axioplan fluorescent microscope, equipped with an apotome, revealed the presence of strong positive signals (green) for GPR109A in the basolateral membrane of RPE (Figure 3). Positive signals for MCT1 (red) were associated with the apical membrane of this cellular layer as expected. Merging of positive signals for GPR109A and MCT1 did not result in any significant overlap, suggesting that these two proteins do not colocalize, and that GPR109A is localized to the basolateral membrane of the RPE.

### Analysis of GPR109A expression in cultured retinal cells

**Figure 4:**
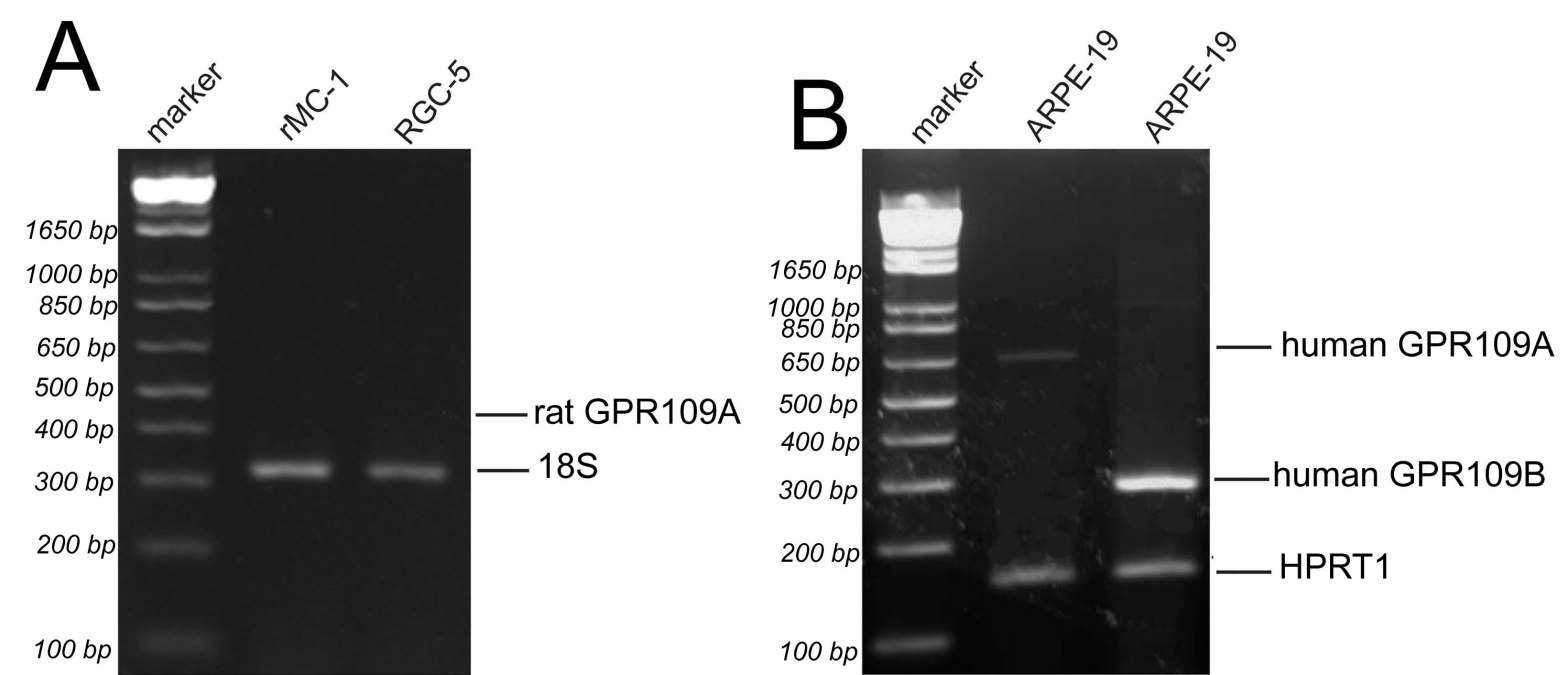
RT–PCR analysis of GPR109A expression in transformed retinal cell lines. **A:** RT–PCR of RNA samples collected from rat Müller (rMC-1) and rat ganglion (RGC-5) cells using PCR primers specific for rat GPR109A did not detect the presence of mRNA transcripts encoding GPR109A. 18S rRNA was used as an internal control. **B**: RT–PCR of RNA samples collected from ARPE-19 cells, using primer pairs specific for human GPR109A and GPR109B respectively, detected the presence of mRNA transcripts for both isoforms.

Immunofluorescence analysis of cryosections of intact mouse retina demonstrated localization of GPR109A protein exclusively in RPE, specifically in the basolateral membrane. To confirm the cell type-specific expression of GPR109A protein in the retina, we used RT–PCR to detect GPR109A mRNA in three transformed retinal cell lines: rMC-1, RGC-5, and ARPE-19 (Figure 4). No product was detectable with RNA prepared from rMC-1 and RGC-5 cells (Figure 4A), confirming the data from the previously described experiments that Müller cells and retinal ganglion cells do not express the receptor. However, RT–PCR with RNA from ARPE-19 cells amplified a product of the expected size (650 bp) for GPR109A. Since ARPE-19 is a human cell line, we also evaluated the expression of GPR109B, the second gene that codes for a highly homologous receptor in humans. These studies showed that ARPE-19 cells express not only GPR109A but also GPR109B (actual product size 280 bp). The molecular identity of the PCR products obtained from ARPE-19 cells was confirmed by sequencing.

**Figure 5:**
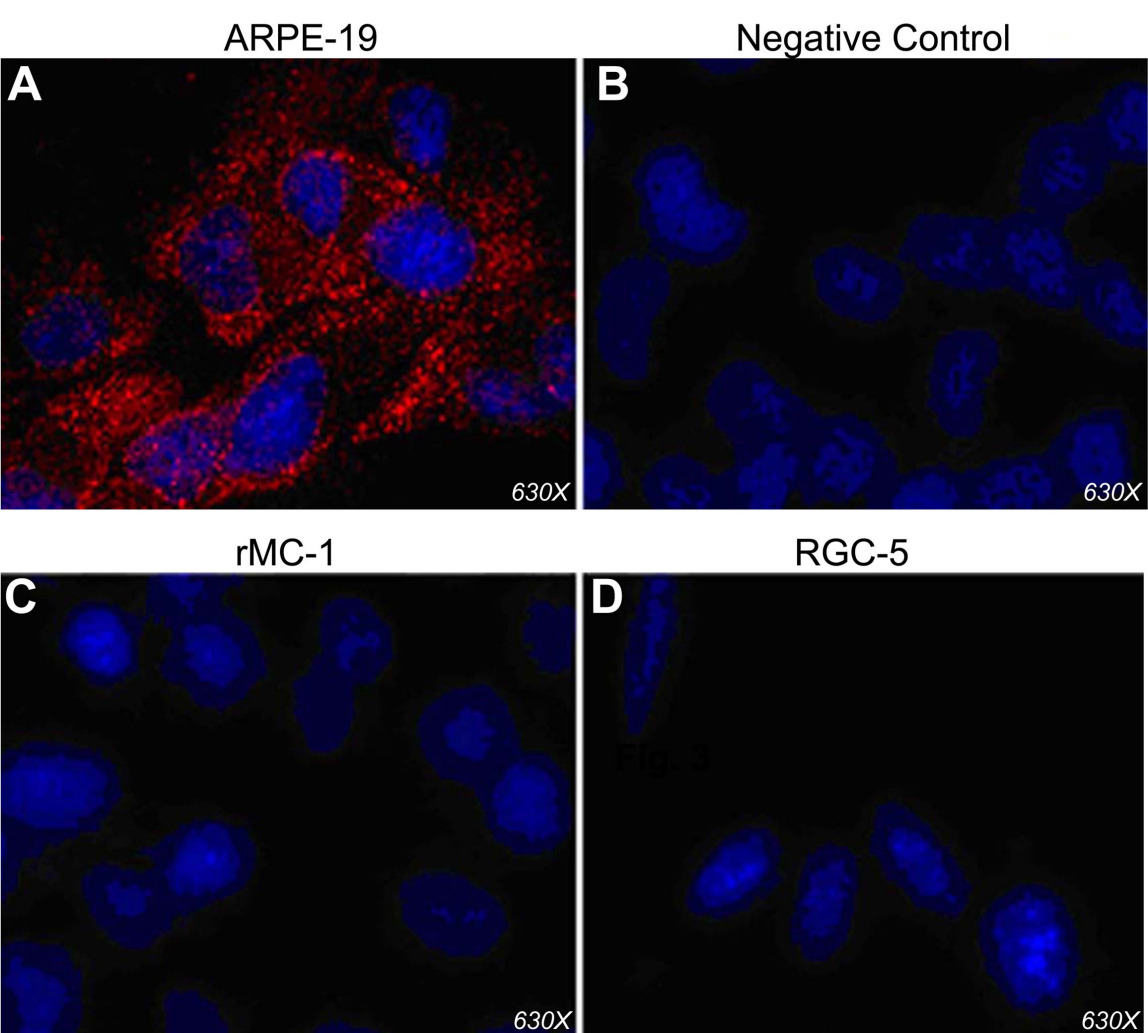
Immunofluorescence analysis of GPR109A protein in ARPE-19, rMC-1, and RGC-5 cells. **A:** ARPE-19 cells incubated with anti-GPR109A antibody, which detects both GPR109A and GPR109B, were highly positive. **B:** Cells incubated with primary GPR109A antibody neutralized by incubation with excess antigenic peptide did not display any positive signal. Likewise, no positive signal was detected in rat Müller (rMC-1; **C**) and retinal ganglion-5 (RGC-5; **D**) cells incubated with anti- GPR109A antibody.

These results showing RPE-cell-specific expression of GPR109A mRNA were corroborated with data from immunofluorescence analysis of the receptor protein (Figure 5). ARPE-19 cells incubated with GPR109A-specific antibody were highly positive as indicated by the strong red fluorescent signal (Figure 5A). Hoechst 33342 counterstaining of the cell nuclei is shown in blue. Immunofluorescence analysis of ARPE-19 cells incubated with peptide-blocked primary GPR109A antibody (negative control) revealed no positive signals (Figure 5B). It has to be noted here that the antibody used will not differentiate between GPR109A and GPR109B in ARPE-19 cells. There were no detectable positive signals for GPR109A protein in rMC-1 (Figure 5C) or RGC-5 (Figure 5D) cells. Since rodents have a single gene coding for GPR109A, absence of immunopositive signals in rMC-1 and RGC-5 cells show that the receptor is not expressed in these cells.

### Analysis of GPR109A expression in mouse primary RPE cells

**Figure 6:**
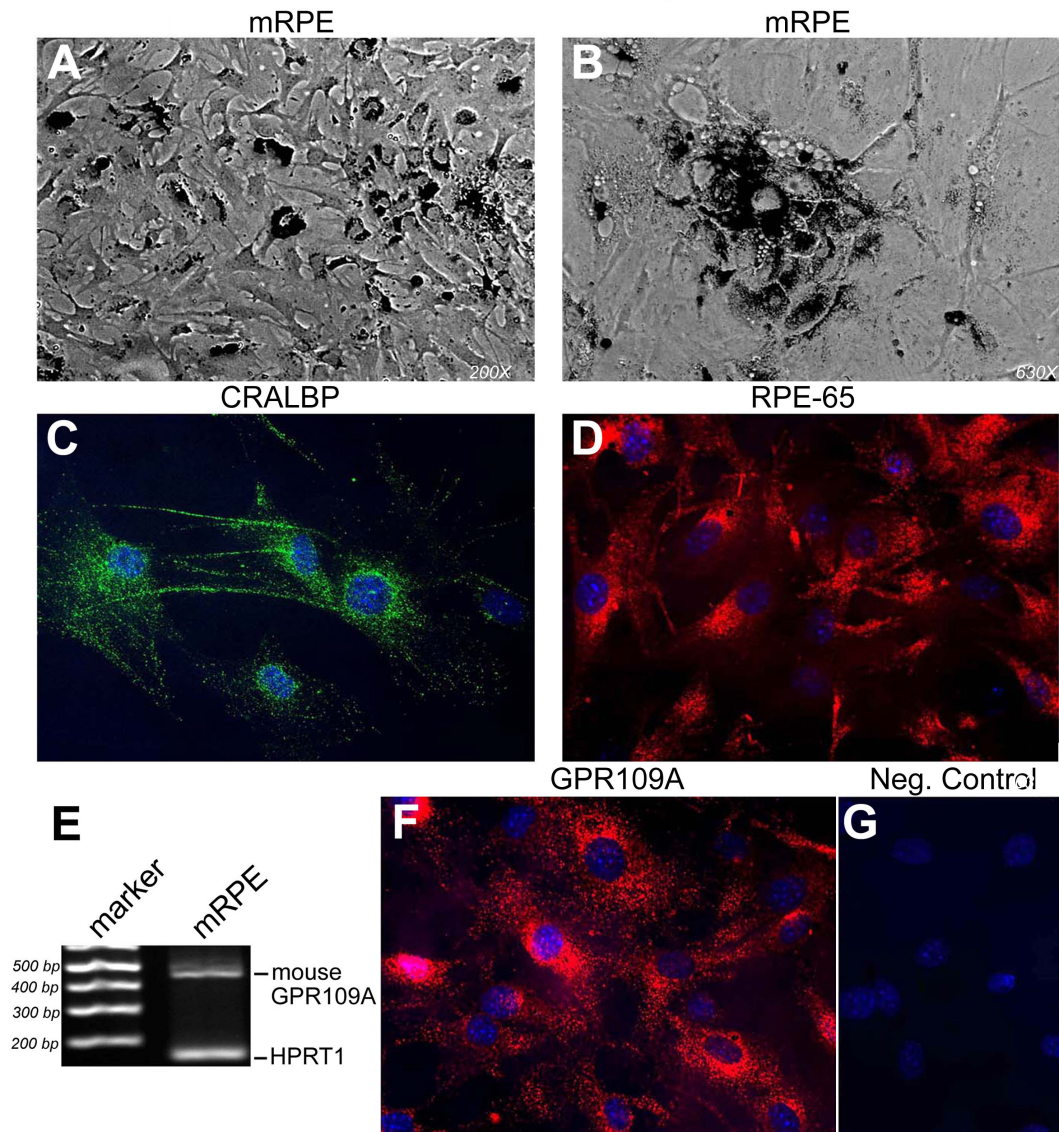
Analysis of GPR109A mRNA and protein expression in primary RPE cells isolated from mouse retina. **A:** This phase-contrast image is representative of the morphology of mouse primary retinal pigment epithelium (mRPE) after 6 days in culture. **B:** In this panel, the morphology of mRPE cells is shown at higher magnification. **C:** mRPE cells incubated with antibody against CRALBP were positive for this RPE/Müller cell marker. **D:** mRPE cells incubated with antibody against the RPE-specific cellular marker, RPE-65, were highly positive. **E:** RT–PCR analysis of RNA collected from mRPE cells detected robust GPR109A mRNA expression. **F:** Incubation of mRPE cells with anti-GPR109A antibody yielded positive signals. **G:** No signal was detected in mRPE cells incubated with primary GPR109A antibody neutralized by incubation with excess antigenic peptide.

RT–PCR and immunofluorescence studies of GPR109A expression in transformed retinal cell lines provided further evidence for the expression of the receptor exclusively in RPE. However, interpretation of the data from ARPE-19 cells is subject to limitations. First, these cells are transformed and, therefore, there is no guarantee that the results from this cell line are extrapolatable to normal RPE cells. Second, ARPE-19 cells are of human origin and thus are expected to express both GPR109A and GPR109B. This was evident from the RT–PCR data. Immunofluorescence studies showing positive signals with GPR109A antibody in ARPE-19 cells are difficult to interpret because the antibody would not differentiate between GPR109A protein and GPR109B protein. In this regard, rodents offer a fortuitous model system in which to study GPR109A expression/function, as they only have a single gene coding for the receptor. Therefore, we analyzed GPR109A expression in primary cultures of RPE cells isolated from mouse eyes (mRPE). Prior to analyzing GPR109A expression in these cells, we evaluated the purity of the cells by morphology and by analyzing the expression of various RPE-specific cell markers (Figure 6). Phase-contrast microscopic analysis of the cell preparations after 6 days in culture indicated that the cells had large spherical nuclei and contained numerous dark deposits, suggestive of the presence of pigment granules (Figure 6A). Analysis of the cells at higher magnification after several days in culture also indicated that in addition to displaying robust pigmentation, cultures of these cells also begin to exhibit the classic cobblestone morphology characteristic of RPE cells in culture (Figure 6B). Using immunofluorescence methods, we analyzed the expression of CRALBP, a protein that is considered to be a marker for RPE cells (Figure 6C). The cells were highly positive for CRALBP. However, as retinal Müller cells are also known to express CRALBP, the RPE cultures were analyzed also for expression of RPE-65, a RPE-specific marker (Figure 6D). Hoechst 33342 dye was used to counterstain all cell nuclei. Greater than 95% of cells were positive for RPE-65 protein expression. Immunofluorescence for the neuronal marker MAP2 was not positive (data not shown). Taken together, these data suggest that the method used to isolate these cells yielded a highly pure population of RPE cells.

To determine whether mRPE cells express GPR109A, total RNA was collected from mRPE cells and used for RT–PCR analysis (Figure 6E). Indeed, mRPE cells were found to express GPR109A as evidenced by amplification of a 443 bp product. HPRT was used as an internal control in the PCR reaction. The molecular identity of the PCR product was confirmed by sequencing. Immunofluorescence techniques were then used to analyze GPR109A protein expression in these cells. Cells incubated with primary GPR109A antibody were highly positive (Figure 6F). No positive signal was detected in mRPE cells incubated with pre-immune rabbit serum in place of primary antibody (Figure 6G).

### Functional analysis of HM74A expression in ARPE-19 cells

**Figure 7:**
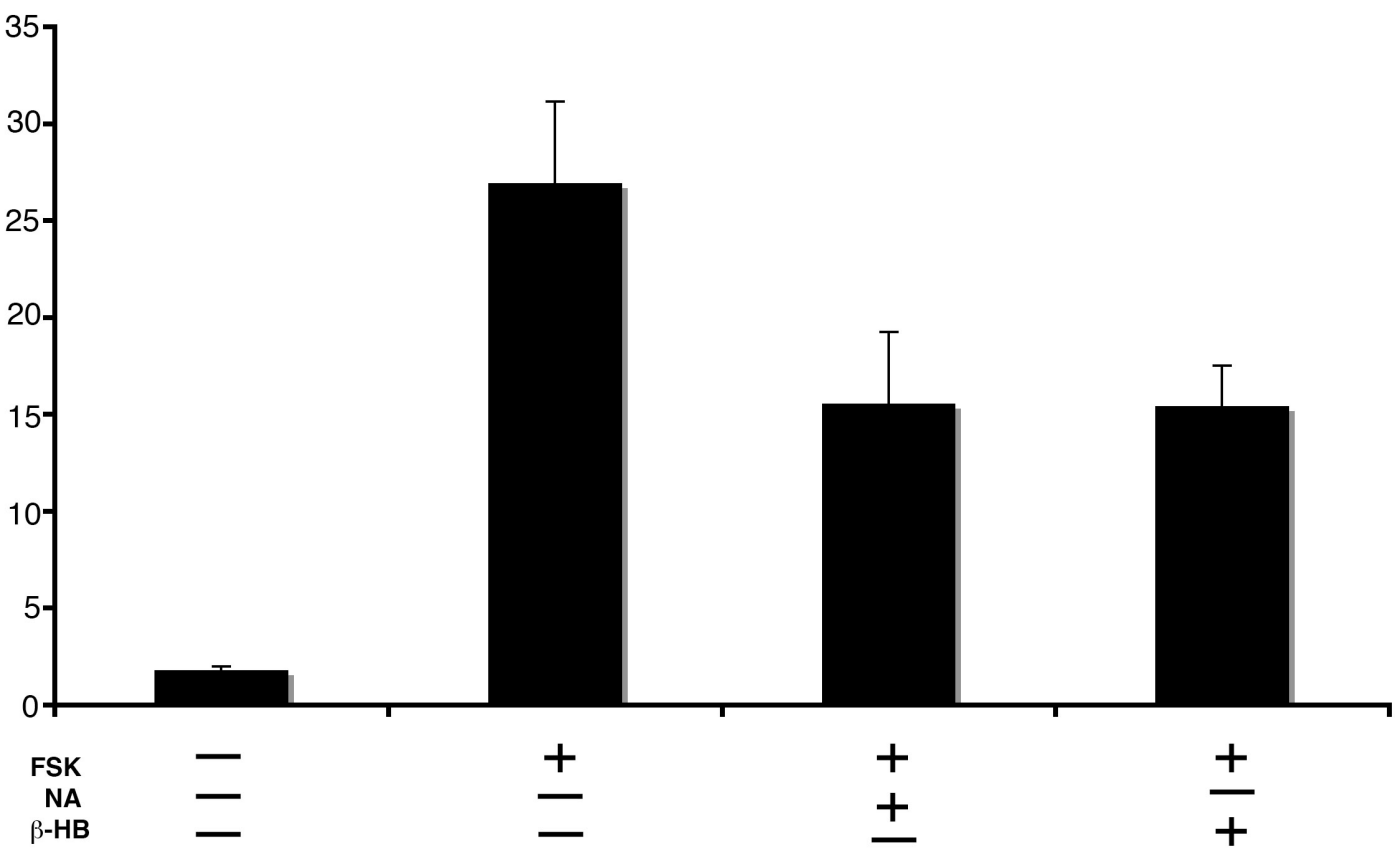
Functional analysis of GPR109A expression in ARPE-19 cells. ARPE-19 cells were treated in the presence or absence of 10 μM forskolin,1 mM nicotinic acid, or 1 mM β-hydroxybutyrate (β-HB) for 1 h followed by measurement of intracellular cAMP levels. Cells incubated in the presence of forskolin alone showed a large increase in intracellular levels of cAMP. This increase was reduced by the addition of nicotinic acid or β-HB to the culture medium, indicating activation of GPR109A. Data are mean±SEM from three separate experiments.

Analysis of GPR109A mRNA and protein revealed expression of the receptor in RPE cells. To determine whether GPR109A receptors expressed in these cells were functional, we assessed the ability of ligands for GPR109A to decrease intracellular cAMP levels as demonstrated in other cell types [5,27]. Treatment of mRPE cells with forskolin increased the cellular levels of cAMP (Figure 7). Treatment of cells with forskolin in the presence of the GPR109A ligands, nicotinic acid or β-HB, decreased cAMP levels, suggesting that GPR109A receptors expressed in RPE are functional.

## Discussion

To date, GPR109A expression is thought to be restricted to adipocytes and immune cells. The antilipolytic activity of niacin is explainable by the presence of the receptor on adipocytes; the biologic role of the receptor on immune cells remains to be determined. Recently, β-HB was found to be the physiologic ligand for the receptor. Patients with diabetes often have marked elevated levels of this metabolite in circulation. These factors combined with our ongoing interest in the retina (particularly diabetic retinopathy) prompted us to ask whether GPR109A is expressed in retina. Prior to the present study, expression of this receptor in retina has not been reported in the literature.

Analysis of GPR109A expression in intact retina by RT–PCR and FISH demonstrated the presence of GPR109A mRNA exclusively in RPE. These expression data were corroborated by immunofluorescence analysis of GPR109A protein. As RPE is a polarized cell with distinct apical and basolateral membranes, further studies were devoted to determining whether GPR109A expressed in the RPE cell layer distributes preferentially to one or both of these distinct membrane compartments. To address this issue, we employed double-labeling immunofluorescent techniques using a marker specific for RPE apical membrane (MCT1). These studies demonstrated unequivocally that in mouse retina, GPR109A protein preferentially distributes to the basolateral membrane in RPE. Data obtained using intact mouse retinal sections were verified by examining GPR109A mRNA and protein expression in cultured RPE (ARPE-19), ganglion (RGC-5), and rMC-1 (Müller) cells. Analysis of GPR109A mRNA and protein in these cells demonstrated expression of the receptor only in ARPE-19 cells. Because ARPE-19 cells are of human origin, the cells expressed not only GPR109A but also GPR109B. Since GPR109A and GPR109B exhibit 96% amino acid sequence identity, it is not possible to generate antibodies, which would recognize only GPR109A in human tissues. Only GPR109A functions as a high-affinity receptor for nicotinate. In contrast, GPR109B does not recognize nicotinate as a ligand. The expression of GPR109A in RPE was further confirmed with mouse primary RPE cells as rodents have only a single gene coding for the receptor.

The data presented in the present report demonstrate for the first time the expression of GPR109A in the retina and, even more interesting and important, the restriction of its expression to RPE where the receptor localizes to the basolateral membrane. These findings provide the basis for future studies designed to determine the functional relevance of GPR109A expression in retina. The localization of the receptor to the RPE basolateral membrane places the receptor in a location conducive to its exposure to substrates in the choroidal circulation. Binding of ligand to the GPR109A is associated with decreases in cAMP levels within the cell [5,8]. In the present study, we found this to be true also in RPE cells.

In addition to its roles in the phagocytic turnover of photoreceptor cells, and maintenance of the outer blood-retinal barrier, the RPE cell layer is obligatory for the supply of nutrients to the outer retina, and cAMP is an important regulator of this process [15]. Thus, alterations of cAMP levels in this cellular layer have tremendous implications in terms of nutrient transport and overall neuronal health and retinal function. Under normal physiologic conditions, the GPR109A may have little relevance because of low levels of β-HB or nicotinate in circulation; however, in conditions such as uncontrolled type 1 diabetes, when circulating levels of β-HB are elevated to levels that could have a maximal impact on receptor activity, the expression of the GPR109A in the basolateral membrane of RPE assumes great significance [18].

Furthermore, β-HB may not be the only physiologic ligand for the receptor. Since this metabolite becomes relevant only under conditions such as prolonged starvation and uncontrolled diabetes, in terms of activation of GPR109A, it would mean that the receptor does not have any biologic relevance under normal conditions. This seems unlikely. We speculate that there may be other ligands, not yet identified, that may activate the receptor even under conditions when the levels of β-HB are not elevated. Thus, our discovery that GPR109A is expressed in retina, particularly in the basolateral membrane of the RPE, has important physiologic and pathologic implications.

## References

[1] Vassilatis DK, Hohmann JG, Zeng H, Li F, Ranchalis JE, Mortrud MT, Brown A, Rodriguez SS, Weller JR, Wright AC, Bergmann JE, Gaitanaris GA (2003). The G protein-coupled receptor repertoires of human and mouse.. Proc Natl Acad Sci USA.

[2] Offermanns S (2006). The nicotinic acid receptor GPR109A (HM74A or PUMA-G) as a new therapeutic target.. Trends Pharmacol Sci.

[3] Wigglesworth MJ, Wolfe LA, Wise A (2006). Orphan seven transmembrane receptor screening.. Ernst Schering Found Symp Proc.

[4] Soga T, Kamohara M, Takasaki J, Matsumoto S, Saito T, Ohishi T, Hiyama H, Matsuo A, Matsushime H, Furuichi K (2003). Molecular identification of nicotinic acid receptor.. Biochem Biophys Res Commun.

[5] Tunaru S, Kero J, Schaub A, Wufka C, Blaukat A, Pfeffer K, Offermanns S (2003). PUMA-G and HM74 are receptors for nicotinic acid and mediate its anti-lipolytic effect.. Nat Med.

[6] Wise A, Foord S, Fraser N, Barnes A, Elshourbagy N, Eilert M, Ignar D, Murdock P, Steplewski K, Green A, Marshall F, Wilson S, Pike N (2003). Molecular identification of high and low affinity receptors for nicotinic acid.. J Biol Chem.

[7] Carlson LA (2005). Nicotinic acid: the broad-spectrum lipid drug. A 50th anniversary review.. J Intern Med.

[8] Taggart AK, Kero J, Gan X, Cai T-Q, Cheng K, Ippolito M, Ren N, Kaplan R, Wu K, Wu T-J, Jin L, Liaw C, Chen R, Richman J, Connolly D, Offermanns S, Wright SD, Waters MG (2005). (D)-Beta-Hydroxybutyrate Inhibits Lipolysis via the Nicotinic Acid Receptor PUMA-G.. J Biol Chem.

[9] Benyó Z, Gille A, Bennett CL, Clausen BE, Offermanns S (2006). Nicotinic acid-induced flushing is mediated by activation of epidermal langerhans cells.. Mol Pharmacol.

[10] Schaub A, Futterer A, Pfeffer K (2001). PUMA-G, an IFN-gamma-inducible gene in macrophages is a novel member of the seven transmembrane spanning receptor superfamily.. Eur J Immunol.

[11] Jampol LM (1988). Niacin maculopathy.. Ophthalmology.

[12] Millay RH, Klein ML, Illingworth DR (1988). Niacin maculopathy.. Ophthalmology.

[13] Gass JD (2003). Nicotinic acid maculopathy. 1973.. Retina.

[14] Kannan R, Stolz A, Ji Q, Prasad PD, Ganapathy V (2001). Vitamin C transport in human lens epithelial cells: evidence for the presence of SVCT2.. Exp Eye Res.

[15] Ganapathy V, Ramamoorthy JD, Del Monte MA, Leibach FH, Ramamoorthy S (1995). Cyclic AMP-dependent up-regulation of the taurine transporter in a human retinal pigment epithelial cell line.. Curr Eye Res.

[16] Rodger J, Goto H, Cui Q, Chen PB, Harvey AR (2005). cAMP regulates axon outgrowth and guidance during optic nerve regeneration in goldfish.. Mol Cell Neurosci.

[17] Sakai T, Yoshitoshi T, Nagai Y, Kitahara K (2006). Increased glutamate uptake and GLAST expression by cyclic AMP in retinal glial cells.. Graefes Arch Clin Exp Ophthalmol.

[18] Mitchell GA, Kassovska-Bratinova S, Boukaftane Y, Robert MF, Wang SP, Ashmarina L, Lambert M, Lapierre P, Potier E (1995). Medical aspects of ketone body metabolism.. Clin Invest Med.

[19] Joussen AM, Poulaki V, Le ML, Koizumi K, Esser C, Janicki H, Schraermeyer U, Kociok N, Fauser S, Kirchhof B, Kern TS, Adamis AP (2004). A central role for inflammation in the pathogenesis of diabetic retinopathy.. FASEB J.

[20] Nagai N, Izumi-Nagai K, Oike Y, Koto T, Staofuka S, Ozawa Y, Yamashiro K, Inoue M, Tsubota K, Umezawa K, Ishida S (2007). Suppression of diabetes-induced retinal inflammation by blocking the angiotensin II type 1 receptor or its downstream nuclear factor-kappa B pathway.. Invest Ophthalmol Vis Sci.

[21] Gnana-Prakasam JP, Martin PM, Mysona BA, Roon P, Smith SB, Ganapathy V (2008). Hepcidin expression in mouse retina and its regulation via lipopolysaccharide/Toll-like receptor-4 pathway independent of Hfe.. Biochem J.

[22] Martin PM, Gnana-Prakasam JP, Roon P, Smith RG, Smith SB, Ganapathy V (2006). Expression and polarized localization of the hemochromatosis gene product HFE in retinal pigment epithelium.. Invest Ophthalmol Vis Sci.

[23] Mullis KB, Faloona F (1987). Specific synthesis of DNA in vitro via a polymerase catalyzed chain reaction.. Methods Enzymol.

[24] Stoflet ES, Koeberl DD, Sarkar G, Sommer SS (1988). Genomic amplification with transcript sequencing.. Science.

[25] Philp NJ, Yoon G, Grollman EF (1998). Monocarboxylate transporter MCT1 is located in the apical membrane and MCT3 in the basal membrane of rat RPE.. Am J Physiol.

[26] Philp NJ, Wang D, Yoon H, Hjelmeland LM (2003). Polarized expression of monocarboxylate transporters in human retinal pigment epithelium and ARPE-19 cells.. Invest Ophthalmol Vis Sci.

[27] Kostylina G, Simon D, Fey MF, Yousefi S, Simon HU (2008). Neutrophil apoptosis mediated by nicotinic acid receptors (GPR109A).. Cell Death Differ.

